# HEADROOM APPROACH TO DEVICE DEVELOPMENT: CURRENT AND FUTURE DIRECTIONS

**DOI:** 10.1017/S0266462315000501

**Published:** 2015

**Authors:** Alan Girling, Richard Lilford, Amanda Cole, Terry Young

**Affiliations:** Institute for Applied Health Research, University of BirminghamA.J.Girling@bham.ac.uk; Division of Health Sciences, University of Warwick; Office of Health Economics; School of Information Systems, Computing and Mathematics, Brunel University

**Keywords:** Headroom analysis, Medical devices, Development decisions

## Abstract

**Objectives:** The headroom approach to medical device development relies on the estimation of a value-based price ceiling at different stages of the development cycle. Such price-ceilings delineate the commercial opportunities for new products in many healthcare systems. We apply a simple model to obtain critical business information as the product proceeds along a development pathway, and indicate some future directions for the development of the approach.

**Methods:** Health economic modelling in the supply-side development cycle for new products.

**Results:** The headroom can be used: initially as a ‘reality check’ on the viability of the device in the healthcare market; to support product development decisions using a real options approach; and to contribute to a pricing policy which respects uncertainties in the reimbursement outlook.

**Conclusions:** The headroom provides a unifying thread for business decisions along the development cycle for a new product. Over the course of the cycle attitudes to uncertainty will evolve, based on the timing and manner in which new information accrues. Within this framework the developmental value of new information can justify the costs of clinical trials and other evidence-gathering activities. Headroom can function as a simple shared tool to parties in commercial negotiations around individual products or groups of products. The development of similar approaches in other contexts holds promise for more rational planning of service provision.

The market for medical devices is distinctively different from those in other sectors. Epidemiological studies can provide unusually precise figures for the limits of demand, and there is an economic framework to value health benefits in many parts of the developed world (1;2). Increasingly, additional service costs are weighed against health gains, measured, for instance, in quality-adjusted life-years (QALYs) using a declared (or implied) monetary equivalent for each unit of health benefit. By integrating this information into business reviews, companies can avoid under-pricing products or prematurely rejecting products that lie above the value-for-money threshold.

Manufacturers need a simple approach to make rapid decisions at the start of product development, but one that can be elaborated for later business reviews. The “Headroom” approach provides a thread that can connect analysis at different stages, providing deterministic rules of thumb early on, probabilistic analyses during the development phases and pricing guidelines in preparation for launching the product. The focus on economic evaluation from a company's perspective (3;4) may be termed “supply-side health economics.”

This study draws together what is known about such methods and considers how the method may be consistently developed in future, by presenting a series of simple examples, chosen for their explanatory properties. The National Institute for Health and Care Excellence (NICE) operates an explicit valuation framework for the NHS in England, which is used here, although the approach applies more generally. Discounting is usually applied (currently 3.5 percent for NICE) to calculate the present value of future health and cost implications of a medical intervention, and in business practice to discount future cash-flows for project valuation. However, discounting does not affect the principles presented here, so it has been ignored in the interests of clarity.

## Headroom Approach

The framework operated by NICE in England balances the health and social care cost (HSCC) of a medical intervention against health benefits, measured in QALYs. When a new treatment displaces an existing treatment, the net-benefit is: 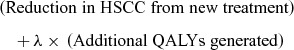 where λ is the threshold for the incremental cost-effectiveness ratio (ICER) which usually lies in the range 20,000 to 30,000 GBP (5). In theory, NICE will recommend any treatment with a positive net benefit but such approval is easier (and quicker) to obtain if it is also cost-saving (5;6)—that is, if HSCC is reduced with no reduction in QALYs—which would entail a value for λ of zero.

In this study, the term “(commercial) headroom” for a new device is the net benefit that would be recognized by the health-care provider if the device were supplied free of charge to the health service. 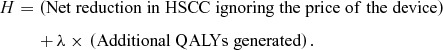 The Headroom, *H*, is the most the manufacturer could charge while securing funding from the care provider—the maximum reimbursable price (MRP)—and sets a ceiling on the unit cost of the new device, including production and development costs. It relies on the same principles as a full economic analysis of a medical intervention but will often be estimated early in the device's development, when data are limited, and so will tend to focus on the more obvious benefits of the relevant therapeutic pathway. This simplification ignores some effects but may provide a useful reality check for the ultimate success of the product.

The idea of headroom analysis was introduced by Sculpher and others (7), using the term “effectiveness gap.” Some examples have been published (8;9), including an iterated approach applied retrospectively across a completed development cycle (10), showing that useful headroom estimates can be obtained even when there is little or no empirical evidence—which is often the case at the outset of development.

## Example: Stapled Hemorrhoidectomy

Stapled hemorrhoidectomy (SH) was introduced into the UK in 1998, and is now recommended for treating 3^rd^ and 4^th^ degree hemorrhoids (11). Chapman et al. (12) conducted a retrospective headroom analysis using information available only up to 1995. SH was proposed as a day-case procedure, so its principal benefit over the standard (Milligan-Morgan) technique was a shorter hospital stay (an estimated saving of 2 days, at 300 GBP/day, in 90 percent of cases), while it was anticipated that SH would accelerate healing and reduce pain (estimated utility-gain of 0.24 for 2 weeks, or 0.24 × 2 ÷ 52 = 0.009 QALYs).

At a 20,000 GBP cost-per-QALY threshold, the resulting headroom estimate is 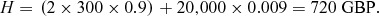


While subject to considerable uncertainty, most (540 GBP) of this headroom derives from a simple cost analysis. By 2007, the stapling device was selling at 420 GBP and recommended by NICE.

Initial development decisions are taken under conditions of greatest uncertainty but they entail the least financial commitment because they can be reversed before significant costs are incurred. Thus, an early decision to abandon a promising idea should not be taken lightly. This supports the recommendation that early stage headroom calculations should adopt “optimistic assumptions in the plausible range.” (8)

By market launch, however, businesses need realistic projections of costs and revenues, so a headroom analysis conducted late in the development cycle should adopt the perspective of a hard-nosed purchaser. Headroom estimates are most useful when regularly reviewed (13) and can also inform the pricing strategy or lead to termination of development.

## Development Decisions

**Figure 1.**
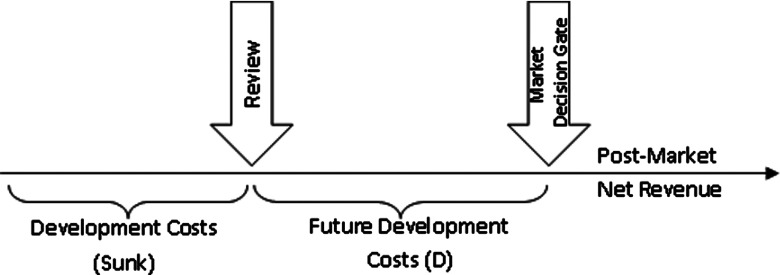
Schematic for a development review of a new device.

Business (or stage-gate) reviews focus on a product's ultimate profitability, and aim to terminate the development where the case cannot be made. At any review (Figure 1), certain costs will have already been committed (sunk costs), so the review must focus on whether the projected market revenues (net of production costs) will cover further development costs (*D*).

Market revenues will often be calculated over a restricted time horizon—perhaps 1 or 2 years—consistent with company policy. Then a simple expression for the value (*V*) of the revenues, net of production costs, is given by: 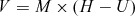 where *M* = the projected number of items sold over the 1- or 2-year horizon, *H* = Headroom estimate of the Maximum Reimbursable Price, and *U* = Estimated cost of production per unit.

However, unless the healthcare provider operates a strict value-based pricing regime (14) it is unlikely that vendors will achieve the full MRP, so it has been argued (15) that some scaling back of sales (reducing *M* by approximately 20 percent) is appropriate, while leaving the rest of the analysis intact.

Even if *V* is negative, uncertainty surrounding the headroom estimate may provide hope of success and a probabilistic approach is warranted when the uncertainty is wide enough to affect the development decision.

## Development Decisions and Uncertainty

Because some design or target performance parameters may be resolved given time and investment, the decision to continue development can be revisited. This insight is the basis of the “real options” approach to business investment (16;17), which recognizes that uncertainty tends to narrow as time goes by, allowing better decisions to be made later and making it less likely that a worthwhile project will be abandoned prematurely.

## Example: Implantable Heart Device

Imagine that a new type of implantable device is proposed for patients with heart failure. The additional healthcare costs are estimated at 3,000 GBP. There are no primary clinical studies in man, and expert opinion is divided on its likely effectiveness. Opinion ranges from those who anticipate a possible average survival gain of approximately 0.5QALYs, to those who fear that the net effect on patients will be negligible. Production costs are estimated at 5,000 GBP per unit.

The situation is summarized in Table 1. We consider three alternative models. Under Model A, we assume that there are two sets of equally credible experts representing opposite extremes of opinion. Depending on which experts you believe, the headroom estimate ranges from minus 3,000 GBP (assuming no health effects) to 0.5 × 30,000 minus 3,000 = 12,000 GBP, based on a Cost-per-QALY threshold of 30,000 GBP. In this case, the best estimate of *H* is the average (−3,000 + 12,000)/2 = 4,500 GBP), which will not cover production costs and suggests abandoning development.

**Table 1.** Economic Models for an Implantable Device Incorporating Uncertainty

		Models for resolvable uncertainty		
Parameter	Estimate (Range)	Model A	Model B	Model C
Extra healthcare costs (per patient)	£3,000 (± £1,000)	point estimate (£3,000)	point estimate (£3,000)	Gaussian: mean £3,000; SD = £500
QALY gain (per patient)	0.25 (± 0.25)	0 or 0.5, (2-point distribution)	Gaussian: mean 0.25; SD = 0.125.	Gaussian: mean 0.25; SD = 0.125.
Value of QALY gain (at £30,000 per QALY)	£7,500 (± £7,500)	0 or £15,000, (2-point distribution)	Gaussian: mean £7,500; SD = £3,750.	Gaussian: mean £7,500; SD = £3,750.
Production Costs (U) (per unit)	£5,000	assumed known	assumed known	assumed known
Adjusted sales projection, year 1 (M)	200 (± 200)	point estimate (200)	point estimate (200)	point estimate (200)

**Figure 2.**
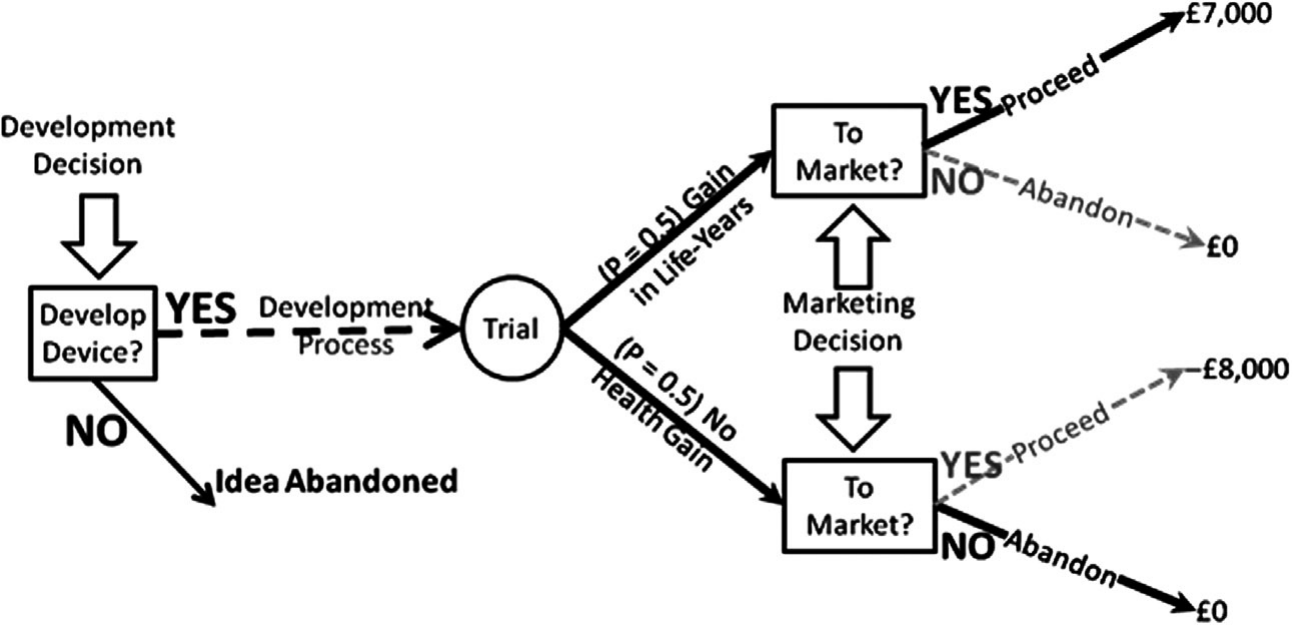
The value of a deferred decision under Model A. The decision to proceed to market will be taken only after the result of the trial is known. The initial estimate of net market-revenues—used to inform the development decision—should take account of the outcome of the later decision and is based only on the outer branches of the decision tree in this example. Assuming equal chances for the two possible trial results this value is ½ × 7,000 + ½ × 0 = 3,500 GBP per item.

However, suppose that development is allowed to proceed until the device can be tested in a clinical trial (see Figure 2). If the pessimists are proved right, development can be terminated before production costs are incurred and postmarket net revenues will be zero. But if the optimists are right and patient survival really is enhanced, the (estimated) net value of postmarket revenues is 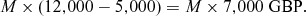 If, at the outset, the experts are equally credible, the best estimate of revenues taking account of the value of the deferred decision, leads to the value: 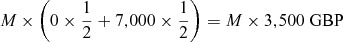 Depending on the market-size, *M*, this may be enough to cover the development costs which, in this example, will include the costs of running the trial.

The impact of a putative clinical trial should be evaluated when making decisions earlier in the development cycle. The same is true for other “developmental uncertainties” (18). For example, there may be initial uncertainty about the cost of production for a new device, which, one anticipates, will be known more accurately by the time a final marketing decision is made. This flexibility is the fundamental justification for using optimistic estimates in an early-stage deterministic headroom analysis (8). It is important to emphasize that optimistic estimates are a substitute for the probabilistic approaches considered here, which should be based on the best estimates available.

Where there is no realistic possibility of resolving uncertainty later in the cycle, there can be no intrinsic value in deferring the decision. This might be because an unfeasibly large study would be required, or because the uncertainty is a function of unknown future market behavior such as the emergence of an alternative treatment (“postmarket uncertainty”).

Table 2 lists some significant parameters according to how susceptible they are to resolution during the product development cycle. Thus, questions of design, functionality, and production costs become clearer once the design is settled. However, market penetration will require knowledge that can only be obtained by going to market. The costs of reaching the market decision gate—the Development Costs, D, in Figure 1—belong to a special category because they will be fully committed before the time of the market-gate review. Best estimates of these costs should therefore be used (perhaps with a sensitivity/scenario analysis) when taking the development decision.

**Table 2.**
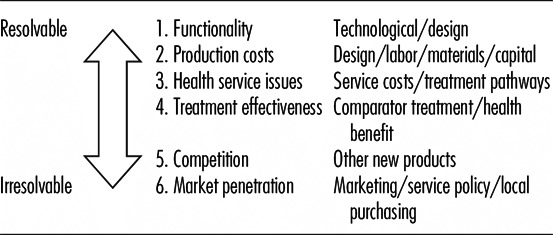
Uncertainties in the Development Cycle

## Value of Resolvable Uncertainty

As information about *H* and *U* accrues, the projected revenue per unit (= *H* – *U*) will be revised, either upward or downward. In the example above, it was suggested that only two outcomes are possible (Table 1, Model A); in practice, it may be more realistic to model future changes in projected revenue using an error distribution as in Model B. The standard deviation (SD), σ, of the distribution reflects the amount of uncertainty which will be eliminated. This translates into extra project value at the interim review (using current estimates of *H* and *U*) equal to 
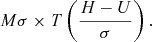


In this expression, *Mσ* is the standard deviation of the possible future changes in projected revenue and *T*(.) is a mathematical function, illustrated in Supplementary Figure 1. for the case of a Normal (Gaussian) error distribution. It is clear from the figure the extra value is greatest when *H = U*, and negligible when (*H – U*) is more than 2σ from zero. The extra value is applied to the project after first working out how much the product would be worth if the marketing decision were made immediately. For the implantable device (Table 1) this “immediate” value is actually zero because the estimate of *H* (4,500 GBP per item) would not justify market launch in the face of estimated production costs of *U* = 5,000 GBP.

Under Model B (Table 1) expert opinion is described by a bell-shaped Normal curve centered on 0.25 QALYs with SD 0.125. At 30,000 GBP per QALY, this translates into a SD of σ = 3,750 GBP for the headroom *H*. So the extra value (per item) from resolving the uncertainty in the QALY-gain is given by 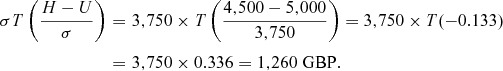 On applying this extra value (multiplied by the first-year sales projection of 200 devices), development should continue provided that *D*, the development cost projection, does not exceed 200 × 1,260 = 252,000 GBP. A further refinement of the approach allows for resolvable uncertainty in healthcare costs (Model C). However, an approximate analysis of this scenario shows a further increase in value of around 1 percent only, and so is ignored here.

In practice, it is unlikely that the health benefit will be fully quantified before the marketing decision is taken and some uncertainty will remain even after a trial, depending on the design and sample-size (19). Thus it may be over-optimistic to identify σ—the SD of the resolvable uncertainty—with the SD of the value of the QALY-gain. A better approach is to set 
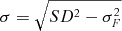
 where σ_F_ is the SD of the QALY-gain that is expected to apply when the trial is complete—a quantity routinely computed for sample size calculations, although usually without taking account of information available before the trial. For example, a trial powered to eliminate half the uncertainty (σ^2^_*F*_ = *SD*^2^/2) would have *σ* = *SD*/√2 and lead, in this example, to an extra value of approximately 830 GBP per item. This is (necessarily) smaller than the earlier figure of 1,260 GBP as calculated above for a comprehensive trial.

## Headroom and Pricing

Manufacturers must calculate *H* just before market-entry, but there is no guarantee that a price set at, or even just below, *H*, will ensure that the healthcare provider buys the product. Thus the headroom estimate is, at best, a prediction for the MRP.

As well as maximizing the chance of reimbursement, price-setting must ensure that the unit costs of production are met and should seek to maximize revenues. This motivates the formulation of price-setting as a formal optimization problem (15), balancing the risk that reimbursement is withheld against the large revenues that accrue when a high price is accepted by the provider. The optimal price will usually lie between *U*, the unit cost of production, and the headroom estimate, *H*, and a lower bound on the optimal price has been identified (= 0.23 × *U* + 0.77 × *H*). This is valid under normally distributed prediction errors and underpins a pragmatic approach to price-setting, where a price of 0.16 × *U* + 0.84 × *H* emerges as close to optimal in many circumstances (15). Even then, there is some chance that reimbursement might be withheld. This chance can be estimated as approximately 5 percent if the prediction standard deviation is 10 percent of the difference between *H* and *U*, leading to a revenue estimate of

0.80 × (*H − U*) per item

This convenient rule of thumb may also be used at earlier stages of the cycle whenever postmarket revenue projections are required.

The estimate may be further reduced by competition or the presence of lower cost devices on the market. For example, one study (10) found an impressive headroom for the use of bio-absorbable pins in the treatment of hallux valgus; yet the simplicity of the device, and the existence of similar low-priced products for other conditions, effectively ruled out the higher price.

## Where Do We Go Next?

Despite the usefulness of the headroom approach it would be idle to pretend that the outlook for a medical product can be reduced to tracking the fortunes of a single numerical quantity as it develops over time. For supply-side decision making, there are important additional criteria including the impact of the new product on the company's product-portfolio and a whole range of competitive issues concerning the positioning of the company in the market place. Nevertheless, at any given time in the development process, a headroom analysis can provide a relatively simple way of maintaining contact with the economic realities of the healthcare market. The situation is not dissimilar to the use of utility-measures by healthcare providers: they do not tell the full story and may need to be supplemented by further equity considerations, but they do provide a framework on which the edifice of technology evaluation can be built.

We now suggest some themes for further development of the approach.

## Practical Commercial Value of the Headroom Method

A validation of assessment methods for new product development opportunities in the medical device sector is complex and largely absent from the literature (20). Recently Chapman et al. (12) used a mixed-methods approach to assess the headroom method's prognostic ability and usability by developers, which involved retrospective application of the method to 20 cases, reporting the sensitivity (92 percent) and negative predictive value (67 percent) according to subsequent NHS uptake. However, these numbers failed to characterize the qualitative information around research and design opportunities revealed by the exercise. Further research could investigate the marginal benefit of additional modelling at the early stages: a commercial application of value of information analysis. Most valuable in extending the current literature base would be a study of the headroom method's use by developers themselves.

Potential users with whom we have discussed the headroom method have identified its utility in negotiation with third parties. For instance, we used an adapted form of the method to demonstrate the payback of a trial of a new version of a device to assist the failing heart muscle (21). Moreover, supply-side methods can inform decisions taken by potential investors, as when a university department seeks to commercialize a discovery. The availability of a shared, simple, tool to both parties in a negotiation can have a profound impact. Simplicity allows many options to be explored quickly during, or between negotiations, while shared information enables both parties to collaborate. The attraction is perhaps even greater where products are bundled or appear in mixed offerings with services. At a time when providers and purchasers are in search of new business models for financial sustainability and are open to profit-sharing and risk-sharing, the use of headroom merits serious and further research.

## Methodological Issues

One of the interesting issues raised when an inventor tries to interest an investor is how to arrive at the probability distributions that provide input data to the model. Work is needed on “calibrating” expert participants so that elicited distributions are neither too optimistic nor too pessimistic. As experience with the use of probabilistic models increases, so increasing amounts of material will accumulate to inform future elicitations. Because considerable uncertainty is a feature of difficult decisions in general, the subject of how to make best use of disparate evidence to make future predictions is a topic that includes, but extends beyond, commercial economics.

Supply-side models also throw into sharp relief a public policy issue when both gains and risks are very large. Such a scenario arises when return on investment covers a very wide distribution of probabilities such that the probability of generating a surplus is low but the returns if a surplus is generated may be massive (as when a scientific investment is of a generic nature). The question then arises as to how risks and returns can be distributed to maximize returns to society. In short, the models we have described to guide commercial decisions may have applications in industrial policy analysis.

## Multi-functional Products

While products used to illustrate this article were fairly typical of those which are assessed by standard health economics, situations arise that are trickier, for instance, where the device has multiple purposes or where its benefits are small but commensurate with low costs per patient. A good example of both issues is the new generation of devices for genetic sequencing that can examine for upward of 100 diseases. For a child with a neurological disorder of unknown cause, there may be a large number of possible diagnoses but, with rare exceptions, no specific medical treatment is available. Patients may, however, benefit from reduced delay in reaching a diagnosis, from simply being able to glean more information, and from identification of other family members who are or are not at risk. It is not easy to capture all possible benefits and harms from such a technology in QALY-type utility values. A willingness-to-pay method, not for each item of benefit and harm, but for the test as a whole, may be a more feasible approach to the valuation of health benefit in such a scenario. The headroom method is easily adapted to substitute such a measure of benefit for utilities, but we know of no examples where this has been done.

## Value Based Pricing

Health service procurement is increasingly moving from a situation where the supplier sets a price which the purchaser must simply decline or accept, to one where the purchaser negotiates a price based explicitly on health benefit (22;23). This method of reimbursement has a subtle implication for pricing when supply-side calculations are made. Under the pricing model explicated above the company's optimal nominated price is accompanied by a risk that reimbursement will be withheld; this price will not necessarily be the lowest price that the company concerned may consider worthwhile. Under a value-based pricing regime the company can obviate the risk by accepting the price offered, but must forgo any additional revenue that may have attached to the higher price they might have sought under the earlier system. Along with the possibility that companies may have reason to be more (or less) optimistic about the final price under a negotiated settlement, this suggests that the issue of pricing be revisited.

It should also be recognized that considerable uncertainty about the effectiveness and safety of a new device may still exist at the point at which the company wishes to proceed to market, invoking the possibility of cost sharing arrangements whereby further information is collected with a view to price adjustment. Such arrangements blur the distinction between supply- and demand-side health economics and raise a complex set of methodological, policy, and even ethical issues. This is a topic in urgent need of further scientific study and policy analysis.

## Other Applications

The headroom method has applications beyond devices and manufactured products to include innovations in service provision and public health interventions. Here, development frequently takes place in the service itself, rather than “off shore” in a company or university laboratory. However, the two things that distinguish economics at the supply side still apply at the development stage; the possibility of holding an option to further develop or amend the innovation pending further data collection and wide parameter uncertainty at the outset. Application of the methods described in this study are thus relevant to the health economic evaluation of service and public health interventions at their formative stages and this is topic of on-going research (24;25). The field would benefit from further developments in methods to prioritize technology developments for these settings.

## CONCLUSIONS

In this study, we have described the progress that has been made in using a simple method iteratively to improve decision making within manufacturing businesses. Headroom estimates can be used to inform successive business reviews, thus connecting up a series of decisions that must be made before a new device can be brought to market. We describe a pragmatic approach, moving from simple threshold-based decisions in the early stages, to those that rely on distributions of probability over future outcomes. Greater use of such methods and the consistent application by suppliers and their customers in healthcare provision, would greatly improve the ability of both sides to ensure value-for-money in the healthcare market.

Finally, we have suggested some areas where further methodological development would exploit the progress made and contribute even more significantly to the technology market and the provision of health services which is increasingly dependent upon it.

## Supplementary material

For supplementary material accompanying this paper visit http://dx.doi.org/10.1017/S0266462315000501. click here to view supplementary material

## CONFLICTS OF INTEREST

AG and RJL have nothing to disclose. AC is employed by the Office of Health Economics, which receives funding from the Association of the British Pharmaceutical Industry (ABPI). TY is an academic whose research is primarily into healthcare technology, systems, and services. This work was done under the MATCH program, which was sponsored by the EPSRC and included a membership and affiliate scheme with commercial contributions. He was a member of the International Scientific Advisory Committee of the Centre for Transformational Molecular Medicine in The Netherlands, which carried a consultancy fee, and his university has received examination feeds for conducting PhD vivas in the field. Since the end of this MATCH and while this paper was being edited and revised, TY has helped to set up the Cumberland Initiative, another membership organization.
