# Supplementary figures and images for: HEADROOM APPROACH TO DEVICE DEVELOPMENT: CURRENT AND FUTURE DIRECTIONS
Source: Int J Technol Assess Health Care. 2015;31(5):331–8. doi: 10.1017/S0266462315000501 (PMC4762238; doi:10.1017/S0266462315000501)

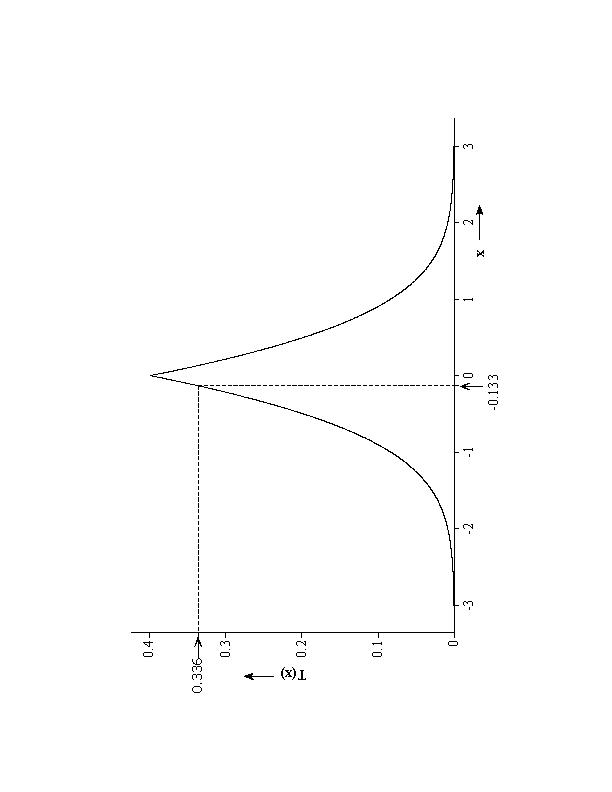

Supplement: Supplementary file 1 [file S0266462315000501sup001.jpg]
